# Supplementary material for: Effects of Herd Establishment Time and Structure on Group-on-Individual Aggression Intensity in Farm Pigs
Source: Animals (Basel). 2024 Jul 31;14(15):2229. doi: 10.3390/ani14152229 (PMC11310940; doi:10.3390/ani14152229)
Supplement: Supplementary file 1 [file animals-14-02229-s001.zip › animals-3048564-supplementary.pdf]

Supplementary Materials for

Effects of Herd Establishment Time and Structure on Group-on-Individual Aggression Intensity in Farm Pig

Zhen Wang [1], Zhengxiang Shi [1] *, Hao Li [1], Hui Liu [1], Zhaowei Xiao [1] Hao Wang [2] and Shihua Pu [2,3]

[1] College of Water Resources & Civil Engineering, China Agricultural University, Beijing 100083, China
[2] National Center of Technology Innovation for Pigs, Chongqing 402460, China
[3] Chongqing Academy of Animal Sciences, Chongqing 402460, China
* Correspondence: shizhx@cau.edu.cn; Tel.: 010-62737570

The file includes:

Text S1.

Tables S1–S8

Data S1–S2

Text S1.

Pilot study. Pigs from different pens were selected and mixed into a larger pen, all at the same time, to form a new herd, H1. There were 20 pigs in H1, all of which were of the Large White × Landrace. Three days later, one pig of the same genetic background was integrated into H1; henceforth, H1 was referred to as the resident herd, with pigs in H1 being referred to as resident pigs. Five days later, another pig of the same genetic background was integrated into H1; each day for the next 15 days, one pig of the same genetic background was integrated into H1. A total of 17 pigs from 17 different pens were integrated into H1. Each integrated pig was unfamiliar with the resident pigs. During the test, we recorded the skin lesions of the integrated pigs. We showed that the level of skin lesions on the integrated pigs was not the same for each integration. Subsequently, we classified the skin lesions of the 17 integrated pigs into 6 levels according to their severity. The levels of skin lesions on the integrated pigs and their definitions are shown in Table S1.

Table S1.

Skin lesions on integrated pigs and their definitions. Based on the severity of the injuries to 17 integrated pigs in the pilot test, we classified their skin lesions into six levels and defined each level.

Skin lesion level	Definition
Slight	Slight skin lesions on individual parts
Minor	Minor skin lesions on individual parts
Mild	Multiple minor skin lesions on individual parts
Moderate	Moderate skin lesions on individual parts
Severe	Severe skin lesions across the whole body
Serious	Serious skin lesions across the whole body

Table S2.

Differences in aggression intensity across herds (CTRL1, TREAT1, CTRL2, and TREAT2). We conducted paired t tests on the differences in AI between groups. Asterisks indicate significant differences between the control and treatment groups (***p<0.01).

Paired number	Item	Mean	SD	MD	*t*	*p*
Paired 1	CTRL1	3.57	1.72	-0.29	-1.549	0.172
	TREAT1	3.86	1.95			
Paired 2	CTRL2	3.29	1.80	-0.43	-2.121	0.078
	TREAT2	3.71	1.80			

Table S3.

Differences in aggression intensity across herds (CTRL1, CTRL2, TREAT3, and TREAT4). We conducted paired t tests on the differences in AI between groups. Asterisks indicate significant differences between the control and treatment groups (***p<0.01).

Paired number	Item	Mean	SD	MD	*t*	*p*
Paired 1	CTRL1	3.57	1.72	1.14	8.000	0.000***
	TREAT3	2.43	1.72			
Paired 2	CTRL2	3.29	1.80	1.14	4.382	0.005***
	TREAT4	2.14	2.12			
Paired 3	CTRL1	3.57	1.72	1.43	7.071	0.000***
	TREAT4	2.14	2.12			
Paired 4	CTRL2	3.29	1.80	0.86	6.000	0.001***
	TREAT3	2.43	1.72			

Table S4.

Nonlinear regression analysis between T and AI. We used a nonlinear parabolic model based on the Levenberg–Marquardt optimization algorithm to perform nonlinear regression analyses of T and AI for the 6 groups.

Variable		CTRL1	CTRL2	TREAT1	TREAT2	TREAT3	TREAT4
A	Value	-0.286	0.714	0.143	-0.429	-0.429	-2.143
	SE	0.380	0.3801	0.481	0.380	0.481	0.65854
	95% CI	-1.341–0.770	-0.341–1.770	-1.192–1.478	-1.484–0.627	-1.764–0.907	-3.971– -0.314
B	Value	0.433	0.115	0.325	0.456	0.198	0.417
	SE	0.073	0.073	0.092	0.073	0.092	0.126
	95% CI	0.231–0.634	-0.087–0.317	0.070–0.580	0.255–0.658	-0.057–0.453	0.067–0.766
C	Value	-0.007	0.007	-0.003	-0.007	0.00265	-0.004
	SE	0.003	0.003	0.004	0.003	0.00374	0.005
	95% CI	-0.015–0.002	-0.002–0.015	-0.013–0.008	-0.015–0.002	-0.008–0.013	-0.018–0.010
R		0.990	0.994	0.995	0.990	0.990	0.987
RMSE		0.309	0.244	0.244	0.309	0.309	0.423
Adj-R^2		0.984	0.982	0.968	0.984	0.968	0.960

Table S5.

Repeated measures ANOVA results (CTRL1, TREAT1, CTRL2, TREAT2 and T).

Effect	Square of sum	Errors	df	Errors	Mean square	Errors	*F*	*p*	Generalized eta-squared	Partial η²
Intercept	364.321	0.357	1	2	364.321	0.179	2040.200	0.000	0.996	0.999
Size	0.893	0.357	1	2	0.893	0.179	5.000	0.155	0.373	0.714
T	77.429	1.143	6	12	12.905	0.095	135.500	0.000	0.981	0.985
Size: T	0.857	1.143	6	12	0.143	0.095	1.500	0.259	0.364	0.429

Table S6.

The result of intra-group effect analysis (T and the number of pens used to form the herds).

T	Pen	MD	SE	*t*	*p*
3	2 and 6	2.000	0.463	4.320	0.012
6	2 and 6	2.000	0.463	4.320	0.012
12	2 and 4	1.500	0.463	3.240	0.041
12	2 and 6	1.500	0.463	3.240	0.041

Table S7.

Contribution rate and utility value of factors affecting aggression intensity. We performed a conjoint analysis of the effects of T (7 levels), number of pens (2 levels), size (2 levels), and genetic background (2 levels) on the AI.

Factor	Contribution	Contribution rate	Level term	Utility value
T	5.167	54.660%	3	-2.667
			6	-1.667
			9	-0.667
			12	0.000
			15	0.833
			18	1.667
			21	2.500
Size	0.714	7.557%	9	-0.357
			12	0.357
Number of pens	3.571	37.783%	2	2.286
			4	-1.000
			6	-1.286

Table S8.

Conjoint analysis estimates of factors affecting aggression intensity.

	Level term	r	SE	*t*	*p*
	Const	3.429	0.164	20.887	0.000
T			12 (reference)		
	3	-2.667	0.205	-13.025	0.000
	6	-1.667	0.205	-8.141	0.000
	9	-0.667	0.205	-3.256	0.003
	15	0.833	0.205	4.070	0.000
	18	1.667	0.205	8.141	0.000
	21	2.500	0.205	12.211	0.000
Size			9 (reference)		
	12	0.357	0.134	2.665	0.012

	Level term	r	SE	*t*	*p*
			2 (reference)		
Number of pens	4	-1.000	0.164	-6.092	0.000
	6	-1.286	0.164	-7.833	0.000

Data S1.

Photographs of skin lesions on integrated pigs and one resident pig. In the TREAT1 group, at T = 18, the number of skin lesions on the integrated pigs had already reached a serious level. Therefore, at T=21, no more pigs were integrated. Further details are presented in the Materials and Methods. Furthermore, note that except for the image of the resident pig in TREAT4 when T=3, all others are pictures of integrated pigs.

T	CTRL1	TREAT1	CTRL2	TREAT2	TREAT3	TREAT4
3	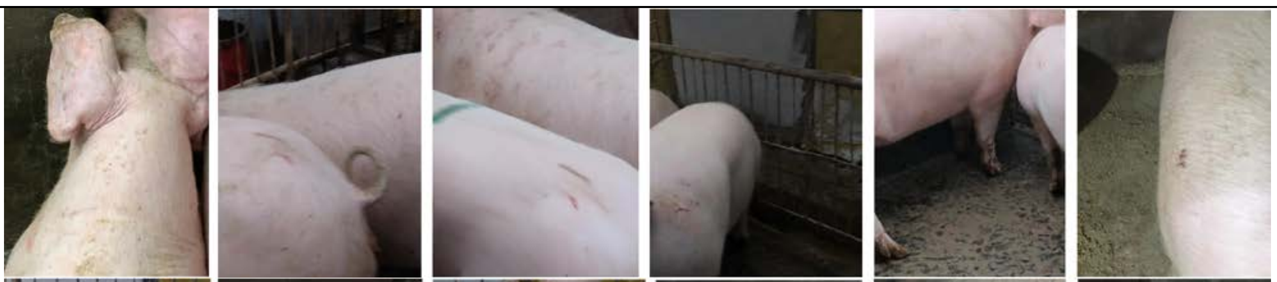					
6	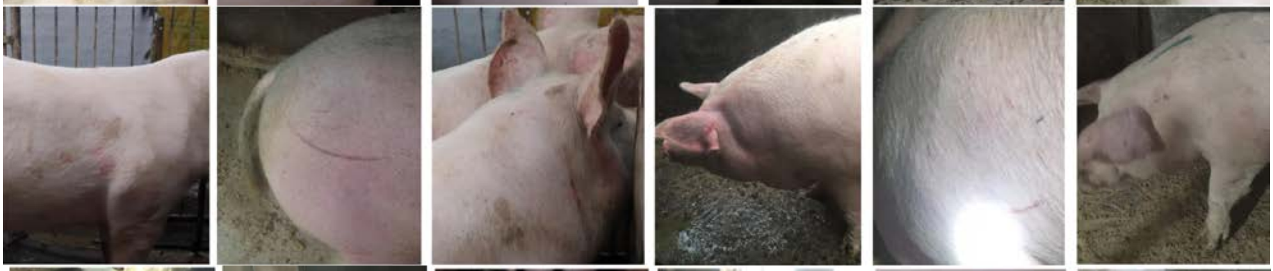					
9	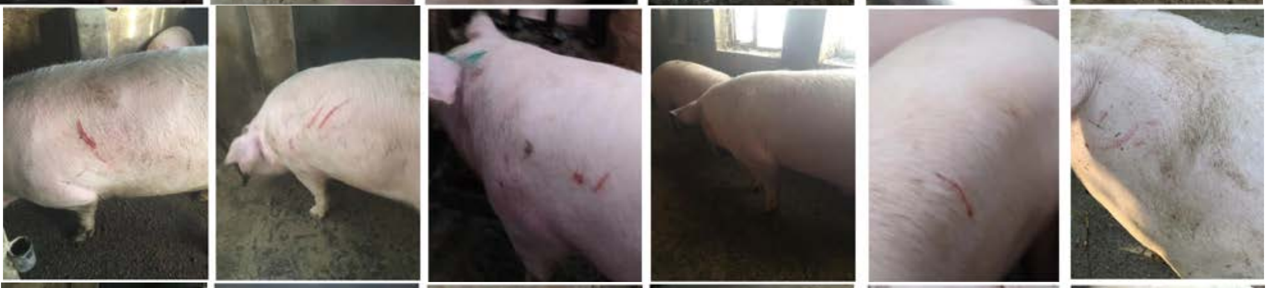					
12	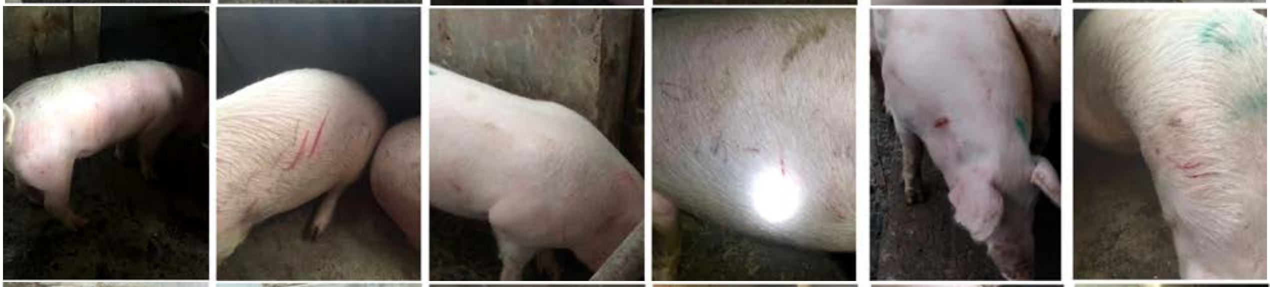					
15	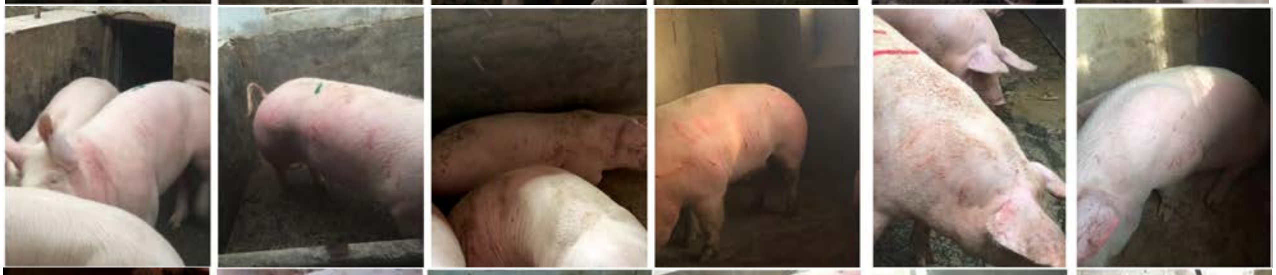					
18	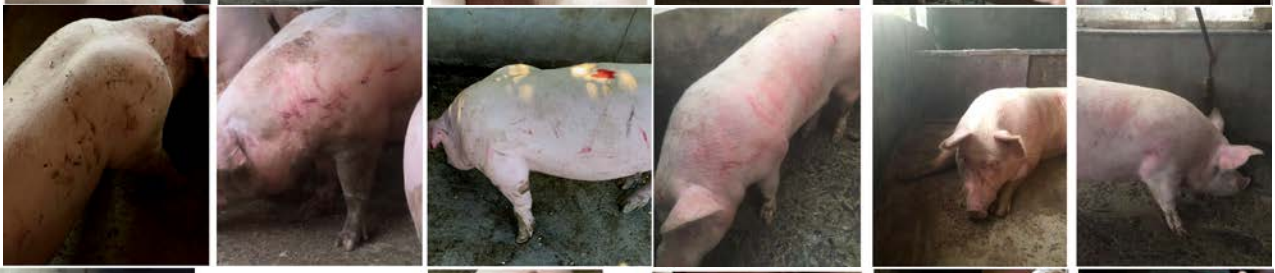					
21	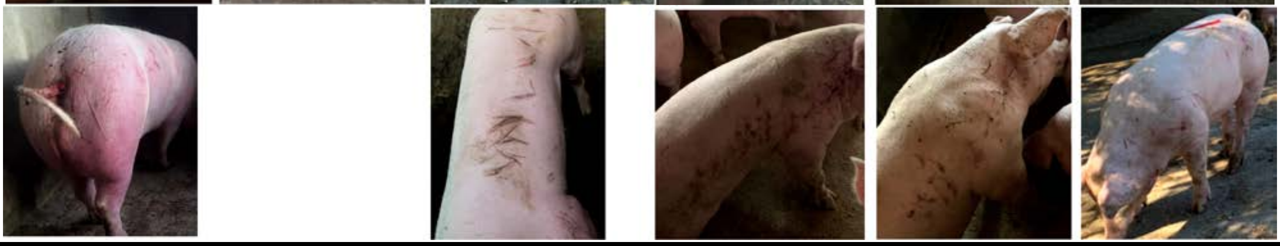					

Data S2.

Aggression intensity scores of resident pigs toward integrated pigs in 6 herds.

T	CTRL1	TREAT1	CTRL2	TREAT2	TREAT3	TREAT4
3	1	1	1	1	0	-1
6	2	2	2	2	1	0
9	3	3	2	3	2	2
12	4	4	3	4	2	2
15	4	5	4	5	3	3
18	5	6	5	5	4	4
21	6	6	6	6	5	5
